# Sexual health literacy as a predictor of reproductive health and family planning attitudes among university students: a cross-sectional study

**DOI:** 10.1093/sexmed/qfag063

**Published:** 2026-07-14

**Authors:** Aysu Yıldız Karaahmet, Fatma Şule Bilgiç, Sevda Karakaş

**Affiliations:** Department of Midwifery, Faculty of Health Sciences, Biruni University, Istanbul, 34100, Turkey; Department of Midwifery, Çanakkale Onsekiz Mart University Faculty of Health Sciences, Çanakkale, 17100, Turkey; Department of Nursing, Faculty of Health Sciences, Gümüşhane University, Gümüşhane, 29100, Turkey

**Keywords:** sexual health literacy, reproductive health, family planning, university students

## Abstract

**Introduction:**

This study was conducted to investigate the relationship between sexual health literacy (SHL) and reproductive health and family planning (RHFP) attitudes among university students and to identify the sociodemographic and informational predictors influencing these variables.

**Methods:**

A descriptive, cross-sectional design was employed with a sample of 867 university students aged 18-35 in Türkiye between May and September 2024. Data were collected using a Sociodemographic Questionnaire, the Sexual Health Literacy Scale (SHLS), and the Reproductive Health and Family Planning Attitude Scale (RHFPAS). Data analysis included Pearson correlation and multiple linear regression models to determine predictors and relationships.

**Results:**

The findings revealed a strong positive correlation between SHL and RHFP attitudes (*r* = 0.880, *P* < .05). Multiple linear regression analysis showed that total SHLS scores and students’ conceptual definitions of sexual health were significant predictors of RHFP attitudes. In particular, the knowledge and attitude subscales of the SHLS were identified as key determinants. Furthermore, residence type, sexual activity status, and information sources were found to significantly influence both SHL levels and RHFP attitudes.

**Discussion:**

SHL is a critical determinant in shaping the RHFP attitudes of young adults. Higher literacy levels directly correlate with more positive attitudes toward reproductive health services, suggesting that targeted educational interventions are necessary to improve health outcomes in this population.

## Introduction

University students are considered to be at risk for adverse sexual and reproductive health (SRH) outcomes such as sexually transmitted infections (STIs) and unintended pregnancies due to the high prevalence of risky sexual behaviors within this age group.^1^^,^^2^ Many sexually active young people experience their first sexual intercourse at an early age, often with insufficient sexual health knowledge. Globally, sexual health problems among young people are associated with negative outcomes such as unintended pregnancies, abortions, sexually transmitted diseases, sexual violence, and abuse. These consequences can have long-term negative effects on young people’s physical, psychological, and social well-being, highlighting the importance of preventive strategies tailored to this population.^1^^,^^3^

Sexual health literacy (SHL) refers to individuals’ ability to access, understand, evaluate, and apply information related to sexuality and sexual health, thereby enabling them to make informed decisions and adopt healthy behaviors.^4^^,^^5^ The World Health Organization emphasizes that SHL plays a critical role in reducing the risk of STIs and preventing unintended pregnancies by facilitating the understanding and use of sexual health information. Therefore, improving SHL is considered one of the fundamental components of comprehensive SRH strategies, particularly those targeting young people.^5^^,^^6^

However, young people from diverse cultural backgrounds encounter various cultural and structural barriers in accessing SRH services, which can lead to negative health outcomes. Culture and social context directly shape young people’s access to sexual health information, their attitudes toward it, and their health-seeking behaviors. Thus, addressing SHL with cultural sensitivity is of great importance. Understanding young people’s sources of sexual health information and their perceptions is critical for developing culturally safe and accessible SRH services tailored to their needs.^7–9^

Family planning (FP), one of the most essential SRH services, plays a critical role in improving reproductive health by preventing unintended pregnancies and reducing excessive fertility.^2^^,^^10^ Previous research has shown that young people with higher SHL levels have more positive attitudes toward reproductive health and family planning (RHFP) and are more likely to use modern contraceptive methods.^5^^,^^11^^,^^12^ These findings suggest that increasing SHL may contribute to the development of positive reproductive health attitudes and more effective use of FP services. Strengthening SHL among university students will not only contribute to individual well-being but also improve reproductive health indicators at the population level. In many conservatives and culturally sensitive societies, where sexuality is still largely perceived as a taboo subject, young people’s access to reliable sexual health information remains limited, creating a fertile ground for misinformation. Therefore, initiatives to enhance SHL are an even more critical necessity in such socio-cultural contexts. This study aimed to examine the relationship between SHL and attitudes toward RHFP among university students, and to determine the sociodemographic and informational factors influencing these attitudes.

In line with this general aim, the following research questions were formulated:

1. What are the SHL levels of university students?
2. What are the attitudes of university students toward family planning and reproductive health?
3. Is there a significant relationship between university students’ SHL and their attitudes towards family planning and reproductive health?
4. Does SHL have a significant effect on university students’ attitudes towards family planning and reproductive health?
5. Do the demographic characteristics of the participants (age, gender, place of residence, information sources) affect their SHL levels and their attitudes towards family planning and reproductive health?

## Methods

### Study design

This cross-sectional study was conducted between May 15, 2024, and September 1, 2024. This study was reported in accordance with the Strengthening the Reporting of Observational Studies in Epidemiology statement for cross-sectional studies.

### Population and sample

The study population consisted of university students aged 18-35 years who were within the reproductive age group. The sample was selected using a non-probability sampling method, specifically the snowball sampling technique. Based on the effect size (correlation coefficient *r* = 0.12) reported by Karaahmet and Bilgiç^13^ the minimum required sample size was calculated as 321 students. The survey link remained active for 6 months, and a total of 865 students participated in the study.

#### Inclusion criteria

- Aged 18 years or older
- Enrolled in a university
- Residing in the study country and holding citizenship
- Single
- Completing the questionnaire in full
- Voluntary participation

#### Exclusion criteria

- Not providing informed consent during the survey process
- Having a diagnosed psychiatric disorder and/or using psychiatric medication
- Having a cognitive impairment

### Data collection tools

Data were collected through Google Forms, and the questionnaire consisted of 3 parts:

***Sociodemographic Information Form:*** Included questions on participants’ age, gender, place of residence, and sources of sexual health information.

***Sexual Health Literacy Scale (SHLS):*** Developed by Areskoug-Josefson et al.^14^ and adapted into Turkish by Işık and Yıldırım.^15^ This 17-item, 5-point Likert-type scale measures SHL across 2 dimensions: sexual knowledge (12 items; scores range 12-60, higher scores indicate higher knowledge) and sexual attitudes (5 reverse-coded items; scores range 5-25, with higher scores indicating more negative attitudes toward sexual health knowledge). In the present study, the Cronbach’s alpha coefficient was 0.91.

***Reproductive Health and Family Planning Attitude Scale (RHFPA):*** Developed by Kökçü,^16^ this 52-item, 5-point Likert-type scale evaluates attitudes toward RHFP. Subscales include attitudes toward unintended pregnancies, STIs, and assisted reproductive methods. In the present study, the Cronbach’s alpha coefficient was 0.89.

### Data collection procedure

Following the necessary approvals, a pilot test was conducted with 30 students to evaluate the clarity and comprehensibility of the questions. Since no revisions were required, the data collection process was initiated. The online questionnaire link was distributed via social media and other communication channels. Eligibility criteria and informed consent information were provided on the first page of the survey. Participants who did not meet the criteria or did not provide consent could not proceed. The survey remained active for 6 months, after which data collection was completed.

### Statistical analysis

The collected data were analyzed using SPSS version 26.0. Descriptive statistics included frequency, percentage, mean, and SD. The normality of numerical variables was assessed with the Kolmogorov–Smirnov test. For comparisons, independent t-tests were applied to 2-group analyses, and ANOVA with post hoc tests was used for comparisons of more than 2 groups. Pearson correlation analysis was performed to assess the strength and direction of linear relationships between variables. A *P*-value <.05 was considered statistically significant.

### Ethical considerations

This study was conducted in accordance with scientific and universal ethical principles. Ethical approval was obtained prior to the data collection process (Arel University Ethics Committee, Date:10 May 2024, No: E-52857131-050.04-621011). Informed consent was obtained from all participants, confirming their voluntary participation in the study. Written informed consent was obtained from all participants prior to data collection, confirming their voluntary participation in the study.

## Results

Descriptive characteristics of the participants are shown in Table 1. The mean age was 29.26 ± 1.15 years for women and 22.48 ± 2.48 years for men. Women had slightly higher RHFPAS total scores than men (2.78 ± 0.39 vs. 2.72 ± 0.71), whereas men scored higher in SHLS total and sexual attitude subscales (3.28 ± 0.81 and 2.94 ± 0.89 vs. 3.20 ± 0.62 and 2.73 ± 0.89, respectively). SHLS knowledge scores were similar across genders. Most participants lived in towns (≈87%), and more than half of the women (55.1%) and two-thirds of the men (67.4%) were sexually active. The majority of women defined healthy sexuality as “a healthy sexual life” (52.5%), while men most often associated it with “sexual and reproductive health problems” (43.6%). The internet and social media were the primary sources of sexual health information, while traditional sources (school, health institutions, books/magazines) were rarely used.

**Table 1:** Distribution of sociodemographic characteristics and sexual health literacy scores (*N* = 867).

**Variables**	**Women (*n* = 360)**		**Man (*n* = 507)**	
	Mean ± SD^*^	**Minimum-maximum**	Mean ± SD^*^	**Minimum-maximum**
**Age**	29.26 ± 1.15	18.00-29.00	22.481 ± 2.48	18.00-29.00
RHFPAS^*^ Total score	2.78 ± 0.39	1.00-5.00	2.72 ± 0.71	1.00-5.00
SHLS^*^ Total score	3.20 ± 0.62	1.00-5.00	3.28 ± 0.81	1.00-5.00
SHLS^*^ Attitude of Sexual	2.73 ± 0.89	1.00-5.00	2.94 ± 0.89	1.00-5.00
SHLS^*^ Information of Sexual	3.40 ± 0.78	1.00-5.00	3.42 ± 0.92	1.00-5.00
	***n***	**%**	***n***	**%**
**Living residence**				
Village	20	4.3	25	4.9
City	29	8.2	42	8.3
Town	310	87.6	440	86.8
**Sexually active**				
Yes	195	55.1	339	67.4
No	165	44.9	170	32.6
**What does sexual healthy?**				
Healthy sexuality	189	52.5	202	39.84
Protection from sexually transmitted diseases	64	17.77	84	16.56
Sexual and reproductive health problems	107	29.72	221	43.58
**The person from whom she first learned about sexuality**				
No one	87	24.16	102	20.11
Family members	43	11.94	32	6.31
Partners	78	21.66	67	13.21
Friends	42	11.66	117	23.07
Internet/magazine	110	30.55	189	37.27
**Where to find information about sexuality**				
From the Internet	119	33.05	227	44.77
From social media	78	21.66	88	17.35
From school	65	18.05	44	8.67
From health institutions	45	12.5	68	13.41
From books/magazines	53	14.72	80	17.77

*Abbreviations: RHFPAS = Reproductive Health and Family Planning Attitude Scale; SHLS = Sexual Health Literacy Scale, SD: Standard Devision.

Group comparisons Table 2 showed that residence, sexual activity, perceptions of healthy sexuality, and sources of information were significantly associated with RHFPAS and SHLS scores. Among women, those living in villages, being sexually active, and defining healthy sexuality as “a healthy sexual life” had significantly higher RHFPAS and SHLS scores (*P* < .05). Women who initially learned about sexuality from the internet/social media had higher SHLS scores, whereas those who currently obtained information from books/magazines or health institutions had the highest RHFPAS and SHLS scores (*P* < .01). Among men, village residents had higher RHFPAS scores, while city residents had higher SHLS scores (*P* < .01). Sexually active men and those who learned from partners or the internet/social media also showed higher scores. Men who currently used books/magazines or health institutions as information sources had the highest RHFPAS and SHLS scores (*P* < .01).

**Table 2:** Comparison of reproductive health and sexual health literacy scores according to demographic characteristics and information sources by gender (*N* = 867).

**Variables**	**Women (*n* = 360)**				**Man (*n* = 507)**			
	RHFPAS^*^ Total score	SHLS^*^ Total score	SHLS^*^ Attitude of Sexual	SHLS^*^ Information of Sexual	RHFPAS^*^ Total score	SHLS^*^ Total score	SHLS^*^ Attitude of Sexual	SHLS^*^ Information of Sexual
**Living residence**								
Village^1^	3.07 ± 0.18	3.03 ± 0.57	3.44 ± 0.29	2.86 ± 0.88	3.22 ± 0.95	3.20 ± 2.84	3.04 ± 1.14	3.26 ± 1.27
City^2^	2.86 ± 0.11	3.22 ± 0.64	3.14 ± 0.63	3.25 ± 1.29	2.84 ± 0.73	3.29 ± 0.78	3.20 ± 0.83	3.32 ± 0.87
Town^3^	2.75 ± 0.39	3.20 ± 0.62	2.64 ± 0.88	3.43 ± 0.78	2.68 ± 0.68	3.28 ± 0.79	2.91 ± 0.89	3.44 ± 0.90
*F* ^*^	5.19	5.21	9.201	4.654	6.28	5.32	2.14	0.768
*P*	***.006***	***.012***	***.000***	***.002***	.007	***.002***	.178	***.001***
Bonferroni	1 > 2 > 3	2 > 3 > 1	1 > 2 > 3	3 > 2 > 1	1 > 3 > 2	2 > 3 > 1	2 > 1 > 3	3 > 2 > 1
**Sexual active**								
Yes	2.78 ± 1.27	3.32 ± 0.60	2.71 ± 0.70	3.58 ± 0.74	2.79 ± 0.72	3.30 ± 0.83	2.90 ± 0.93	3.47 ± 0.92
No	2.77 ± 1.43	3.03 ± 0.82	2.70 ± 0.69	3.17 ± 0.79	2.76 ± 0.69	3.23 ± 0.77	3.03 ± 0.82	3.31 ± 0.89
*t* ^*^	0.063	4.474	0.195	0.271	−0.934	0.955	−1.569	1.854
*P*	***.002***	***.000***	.07	***.000***	***.004***	.340	.067	.066
**What does sexual healthy?**								
Healthy sexuality^1^	2.39 ± 1.26	3.23 ± 0.82	3.54 ± 0.13	3.46 ± 1.28	3.62 ± 1.45	3.43 ± 1.84	3.34 ± 2.14	3.38 ± 0.27
Protection from sexually transmitted diseases^2^	2.26 ± 1.28	3.54 ± 0.13	3.24 ± 1.54	3.25 ± 1.29	2.56 ± 1.43	3.68 ± 1.28	3.24 ± 1.53	3.65 ± 1.43
Sexual and reproductive health problems^3^	2.04 ± 1.64	3.62 ± 0.62	3.32 ± 1.38	3.28 ± 0.45	2.75 ± 1.48	3.46 ± 1.49	2.71 ± 1.29	3.24 ± 0.64
*F* ^*^	5.67	5.21	5..43	2.654	4.65	5.32	2.14	.658
*P*	***.028***	.078	***.031***	***.002***	***.003***	***.034***	***.021***	***.032***
**Bonferroni**	**1 > 2 > 3**	**-**	**1 > 3 > 2**	**1 > 3 > 2**	**1 > 3 > 2**	**2 > 3 > 1**	**1 > 2 > 3**	**2 > 1 > 3**
**The person from whom she first learned about sexuality**								
No one^a^	2.44 ± 1.70	3.28 ± 0.66	2.75 ± 0.96	3.49 ± 0.85	2.79 ± 0.55	3.17 ± 0.76	2.74 ± 0.88	3.35 ± 0.91
Family members^b^	2.32 ± 1.22	3.21 ± 0.53	2.54 ± 0.84	3.48 ± 0.670	2.73 ± 0.60	3.16 ± 0.76	2.83 ± 0.82	3.30 ± 0.89
Partners^c^	2.52 ± 1.11	3.35 ± 0.57	3.09 ± 0.89	3.46 ± 0.55	2.66 ± 0.87	3.57 ± 0.95	3.35 ± 0.95	3.66 ± 1.03
Friends^d^	2.07 ± 1.25	2.96 ± 0.66	2.57 ± 0.96	3.11 ± 0.88	2.68 ± 0.82	3.308 ± 0.87	3.09 ± 0.96	3.39 ± 0.93
Internet/social media^e^	2.79 ± 0.07	3.76 ± 0.69	2.91 ± 1.04	3.22 ± 0..79	2.69 ± 0.71	3.83 ± 0.88	3.14 ± 0.85	3.41 ± 0.97
F^*^	2.431	1.81 7	1.572	1.436	1.97	3.054	5.06	1.92
*P*	***0.032***	***0.02***	0.053	0.070	***0.006***	***0.000***	***0.003***	0.076
Bonferroni	e > c	e > c	-	-	a > b	e > c	c > e	-
**Where to find information about sexuality**								
From the Internet^1^	2.19 ± 1.37	3.63 ± 1.97	2.39 ± 1.66	3.39 ± 1.25	2.56 ± 0.43	3.17 ± 0.76	2.74 ± 0.88	3.32 ± 1.21
From social media^2^	2.44 ± 1.21	3.25 ± 0.57	2.97 ± 1.75	2.78 ± 1.21	3.43 ± 1.21	3.16 ± 0.76	2.83 ± 0.82	3.20 ± 0.32
From school^3^	2.11 ± 1.32	3.36 ± 0.30	3.07 ± 1.13	3.17 ± 1.24	3.36 ± 0.87	3.57 ± 0.95	3.35 ± 0.95	3.34 ± 1.21
From health institutions^4^	2.22 ± 1.17	3.96 ± 0.51	3.01 ± 1.40	3.21 ± 1.12	3.58 ± 1.82	3.308 ± 0.87	3.18 ± 0.96	3.26 ± 1.21
From books/magazines^5^	2.71 ± 1.44	3.07 ± 1.37	3.12 ± 0.36	3.16 ± 0.36	3.69 ± 0.71	3.83 ± 0.88	3.34 ± 0.85	3.51 ± 0.54
*F* ^*^	4.531	4.107	1.130	1.130	3.531	5.107	3.768	0.876
*P*	***0.001***	***0.003***	0.341	0.121	***0.001***	***0.003***	0.078	0.431
Bonferroni	***5 > 2***	***4 > 1***	-	-	**5 > 4**	**5 > 3**	-	-

*Abbreviations: *F* = 1 way ANOVA; RHFPAS = Reproductive Health and Family Planning Attitude Scale; SHLS = Sexual Health Literacy Scale; *t* = Independent *t*-test, Bold, italic: p<0.05.

Pearson correlation analyses (Table 3) indicated a positive association between age and RHFPAS scores (*r* = 0.156, *P* < .001) and a strong positive correlation between SHLS total and RHFPAS scores (*r* = 0.880, *P* < .05). The SHLS knowledge subscale showed a particularly strong association with RHFPAS (*r* = 0.942, *P* < .001), and SHLS attitude was moderately correlated (*r* = 0.600, *P* < .001).

**Table 3:** Pearson correlation coefficients between reproductive health attitudes and sexual health literacy scores (*N* = 867).

**Variables**	**r/p**	**Age**	RHFPAS^*^	SHLS^*^ Total	SHLS^*^ Sexual information	SHLS^*^ Sexual attitude
RHFPAS^*^	*r*	.156	1			
	*P*	.000				
SHLS^*^ Total	*r*	.537	.880	1		
	*P*	.002	.039			
SHLS^*^ Sexual information	*r*	.333	.942	.974	1	
	*P*	***.001***	***.000***	***.021***		
SHLS^*^ Sexual attitude	*r*	.870	.600	.883	.754	1
	*P*	***0.002***	***.000***	***.021***	***.007***	

Pearson Correlation Test. Abbreviations: SHLS = Sexual Health Literacy Scale; RHFPAS = Reproductive Health and Family Planning Attitude Scale, Bold, italic: p<0.05.

Multiple regression analysis (Table 4) was conducted to identify predictors of RHFPAS total scores. SHLS total score and perceptions of healthy sexuality (“What is sexual health?”) were found to be significant predictors when controlling for age, gender, and residence (*F* (5,842) =5.178, *P* < .001; *R*^2^ = 0.030, Adj. *R*^2^ = 0.024). Specifically, SHLS total score (β = −0.140, *P* < .001) and perceptions of healthy sexuality (β = −0.070, *P* = .044) predicted RHFPAS scores in a negative direction, while age, gender, and residence were not significant predictors (*P* > .05).

**Table 4:** Multiple linear regression analysis predicting attitudes toward reproductive health and family planning from sexual health literacy (total score) and demographic variables.

**Dependent variable**	**Independent variable**	**β**	**SE**	**Beta**	***t***	***P***	VIF^*^	***F***	**Model (*P*)**	***R*** ^**2**^	**Durbin** **Watson**
RHFPAS^*^	Constant	3.203	0.135	–	23.776	<.001	–	5.178	<.001	0.024	1.778
	SHLS Total^*^	−0.113	0.028	−0.140	−4.072	<.001	1.022				
	Age	0.000	0.000	0.010	0.280	.779	1.011				
	Gender	−0.055	0.042	−0.045	−1.314	.189	1.023				
	Living residence	0.038	0.043	0.031	0.880	.379	1.057				
	Source of information	−0.019	0.010	−0.070	−2.016	.044	1.036				

*Abbreviations: RHFPAS = Reproductive Health and Family Planning Attitude Scale; SHLS = Sexual Health Literacy Scale; VIF = Variance Inflation Factor.

In a second regression model including SHLS subscales (Table 5), SHLS knowledge negatively (β = −0.219, *P* < .001) and SHLS attitude positively (β = 0.106, *P* = .003) predicted RHFPAS scores, while perceptions of healthy sexuality continued to be a negative predictor (β = −0.084, *P* = .015). Age, gender, and residence remained non-significant. This model was statistically significant (*F* (6,841) =7.960, *P* < .001) and explained approximately 5% of the variance in RHFPAS scores (*R*^2^ = 0.054, Adj. *R*^2^ = 0.047).

**Table 5:** Multiple linear regression analysis predicting attitudes toward reproductive health and family planning from sexual health literacy subscales (knowledge and attitudes) and demographic variables.

**Dependent variable**	**Independent variable**	**β**	**SE**	**Beta**	***t***	***P***	VIF^*^	***F***	**Model (*P*)**	***R*** ^**2**^	**Durbin** **Watson**
RHFPAS^*^	Constant	3.239	0.133	–	24.287	<.001	–	7.960	<.001	0.047	2.078
	Age	0.000	0.000	0.010	0.297	.766	1.011				
	Gender	−0.081	0.042	−0.067	−1.955	.051	1.043				
	Living residence	0.014	0.043	0.011	0.321	.749	1.073				
	Source of information	−0.023	0.010	−0.084	−2.437	.015	1.044				
	SHLS^*^ Sexual attitude	0.070	0.024	0.106	2.942	.003	1.154				
	SHLS^*^ Sexual information	−0.151	0.025	−0.219	−6.097	<.001	1.145				

*Abbreviations: RHFPAS: Reproductive Health and Family Planning Attitude Scale; SHLS: Sexual Health Literacy Scale; VIF, Variance Inflation Factor.

## Discussion

This study aimed to examine the relationship between SHL and attitudes toward RHFP among university students, and to determine the sociodemographic and informational factors influencing these attitudes. This study demonstrated that SHL significantly predicts RHFP attitudes among university students. Students with accurate knowledge adopted healthier attitudes more strongly. Importantly, SHL knowledge and attitudes predicted reproductive health attitudes independent of sociodemographic characteristics, highlighting the potential effectiveness of educational interventions.

In cultures where East–West conflicts around sexuality persist, young women with low awareness of SHL concepts often continue traditional behaviors due to social environment and family pressures.^2^^,^^17–19^ Our findings further revealed that students living in rural areas, those who were sexually active, and those who defined sexual health as “a healthy sexual life” had higher SHL knowledge and more positive RHFP attitudes. This suggests that living conditions and personal experiences may shape health-related attitudes. Supporting this, a study among Iranian women reported low levels of SRH knowledge,^12^ while another study with university students found no association between place of residence and SHL.^19^

Understanding how cultural and social contexts influence SRH and access to care is essential.^7^ In the present study, male students’ SHL knowledge and attitudes were higher in urban areas, while their RHFP attitudes were more positive in rural areas. This divergence may reflect the differential effects of gender norms and opportunities for accessing information. A systematic review conducted in Australia among culturally diverse youth found that sexual health knowledge was low, cultural influences were strong, and access to information was limited. The review emphasized the need for culturally sensitive, equity-oriented strategies that overcome the “culture of silence”.^7^ These findings underscore the importance of not overlooking cultural barriers in access to SRH knowledge and services, and the necessity of developing youth-oriented programs that ensure culturally responsive and equitable access.

SHL is defined as the ability to find, understand, and effectively use information and services needed to guide sexual health-related decisions and behaviors.^20^ In this study, students with higher SHL levels had more positive RHFP attitudes, indicating that accurate knowledge plays a critical role in shaping healthy attitudes. However, a systematic review highlighted that existing SHL measurement tools do not fully capture all dimensions of the construct, pointing to an urgent need for valid, reliable, and culturally adapted instruments.^20^ Evidence from sub-Saharan Africa has shown that sexual health knowledge among young people is alarmingly low, with negative attitudes toward SRH.^21^ Similarly, studies from Iran reported low SHL among women not using contraceptives,^12^ while an umbrella review from Ethiopia revealed high unmet needs for family planning knowledge and services, adversely affecting SHL.^22^ A study among Colombian high school students found that those who received sexual health education demonstrated greater acceptance of family planning methods and higher levels of knowledge.^23^ Another study with young women reported higher SHL levels, more positive attitudes, and lower engagement in premarital risky sexual behaviors.^24^ Collectively, these findings suggest that increasing SHL should not be limited to knowledge transfer but should also involve ensuring access, cultural appropriateness, and comprehensive educational strategies that empower individuals to develop healthier attitudes and behaviors.

The findings of this study provide a robust evidence base for reshaping reproductive health strategies within higher education and clinical nursing practice. At the policy level, the strong correlation (*r* = 0.880) between SHL and reproductive health attitudes necessitates the integration of structured SHL frameworks into national university curricula. Rather than isolated seminars, policy-makers should prioritize the development of multidisciplinary health education standards that address the sociodemographic disparities identified, such as residence type and sexual activity status. Furthermore, establishing low-threshold, anonymous counseling centers within campus health services is essential to ensure that students can translate theoretical literacy into safe, practical health behaviors. Although a strong positive correlation was observed between SHL and RHFP attitudes, the relatively low explained variance in the regression models suggests that these attitudes are likely influenced by multiple psychosocial, cultural, interpersonal, and educational factors beyond SHL alone. Furthermore, the very high correlations observed between the SHLS and RHFPAS, particularly for the knowledge dimension, may reflect conceptual proximity between the constructs measured by the 2 instruments, indicating that greater SHL is closely intertwined with more favorable RHFP attitudes. Therefore, while SHL appears to be an important determinant, future studies should investigate additional contextual and behavioral factors that may contribute to RHFP attitudes among young adults.

In clinical practice, nursing and midwifery professionals should move beyond traditional knowledge-transfer models toward attitude-focused interventions. Given that literacy levels significantly predict family planning attitudes, nurses and midwives can utilize the SHLS as a routine screening tool in primary care to identify at-risk individuals. There is also a critical need for nursing-led digital health initiatives; by creating evidence-based digital platforms, the profession can counteract the widespread misinformation identified in students’ informal information sources. Ultimately, fostering peer-led advocacy programs and “Sexual Health Ambassador” roles within nursing departments can leverage peer influence to bridge the gap between health literacy and positive reproductive health outcomes during the transition to adulthood.

### Strengths and limitations

The strengths of this study include a relatively large sample size, the inclusion of both male and female students, and the use of valid and reliable measurement tools. The application of detailed statistical analyses further enhanced the reliability of the findings. However, the cross-sectional design prevents causal inferences, and reliance on self-reported data may have introduced social desirability or response bias. Moreover, as the sample consisted solely of university students, the findings may not be generalizable to the entire young adult population.

Building upon the findings of this cross-sectional study, future research should prioritize longitudinal designs to establish a clearer temporal relationship between the development of SHL and long-term reproductive health outcomes. While this study identifies a strong correlation between literacy and attitudes, tracking university students into their post-graduate lives would provide invaluable data on how early literacy interventions influence actual family planning choices and clinical health-seeking behaviors over time. Furthermore, given the significant role of information sources identified in this research, there is a clear need for interventional studies that evaluate the efficacy of digital health tools and mobile applications designed specifically to enhance SHL among young adults. Exploring the impact of gamified learning or AI-driven counseling platforms could offer innovative pathways for nursing-led interventions.

Additionally, future investigations should adopt a qualitative or mixed-methods approach to gain a deeper understanding of the sociocultural nuances that shape sexual health definitions and attitudes. Research focusing on the perspectives of diverse subgroups, including international students or those from varied religious and cultural backgrounds, would help in developing more culturally sensitive health literacy models. There is also a significant opportunity for multi-center and cross-cultural comparative studies to determine whether the predictors of reproductive health attitudes found in this Turkish sample are consistent across different European or global contexts. Finally, future research could explore the integration of SHL into the broader framework of “digital health literacy” to better equip nursing professionals with the evidence needed to combat the evolving landscape of online health misinformation.

## Conclusion

This study examined the relationship between SHL and RHFP attitudes among university students. The findings demonstrated that students’ levels of sexual health knowledge and attitudes significantly influenced their RHFP attitudes. In particular, sexual health knowledge emerged as a critical determinant in the development of healthier attitudes.

Residence, sexual activity status, and definitions of sexual health also played important roles. Female students living in rural areas, those who were sexually active, and those who defined sexual health as “a healthy sexual life” scored higher both in SHL and in RHFP attitudes. Among male students, those living in urban areas reported higher SHL knowledge and attitudes, whereas their RHFP attitudes were more positive in rural areas. These findings suggest that access to information and gender norms may shape attitudes in different ways.

Regression analyses further indicated that SHL knowledge and attitudes independently predicted RHFP attitudes, while the way students defined sexual health was also an important determinant. Age, gender, and place of residence were not found to be significant predictors, suggesting that students’ knowledge and perceptions shape their attitudes regardless of demographic characteristics.

In light of these findings, educational interventions aimed at improving SHL among university students may strengthen RHFP attitudes. Training programs should ensure access to accurate information, while considering students’ perceptions of sexual health and their social contexts. Moreover, integrating both modern sources such as the internet and social media with traditional information channels may enhance the effectiveness of these interventions.

## Data Availability

The datasets generated and/or analyzed during the current study are available from the corresponding author on reasonable request, in accordance with ethical and privacy considerations.
